# Estimation of Ewe Live Weight and Carcass Traits Using Advanced Hybrid Deep Learning and Multimodal Feature Fusion

**DOI:** 10.3390/biology15100815

**Published:** 2026-05-21

**Authors:** Ahmad Shalaldeh, Majeed Safa, Chris Logan, Mohmmad Othman

**Affiliations:** 1 Department of Data Science and Artificial Intelligence, Faculty of Information Technology, Al-Ahliyya Amman University, Al Salat 19111, Jordan; 2 Faculty of Agribusiness and Commerce, Lincoln University, Lincoln 7647, New Zealand; majeed.safa@lincoln.ac.nz; 3 Faculty of Agriculture and Life Sciences, Lincoln University, Lincoln 7647, New Zealand; chris.logan@lincoln.ac.nz; 4 School of Health Science, University of Canterbury, Christchurch 8041, New Zealand; mohmmad.othman@canterbury.ac.nz

**Keywords:** self-attention, transformer architecture, hybrid deep learning, ResNet18, multi-layer perceptron, live weight, carcass traits

## Abstract

The non-invasive determination of live weight and body composition of ewes is an important element in ensuring precision livestock management and animal well-being. Traditional practices tend to be subjective, labor-intensive, or rely on expensive medical imaging such as Computed Tomography (CT). This paper proposes a new hybrid deep learning method to predict live weight and carcass traits in Coopworth ewes. The dataset of 1184 images taken from 156 ewes was analyzed and compared using a hybrid model (ResNet18 with Multi-Layer Perceptron through simple concatenation) and two more advanced models: Attention-Guided Feature Fusion Network (AGFF-Net) based on cross-modal attention and a Vision Transformer-based Hybrid Regressor (ViT-HR). Auxiliary tabular variables are the Body Condition Score (BCS) and size category. The Transformer architecture predicts (R^2^ = 0.93) the live weight of ewes by dynamically ranking each visual patch and asking it to query the self-attention sequence. This technique treats the BCS as a distinct token in the self-attention sequence. Data partitioning at the animal level was stringent, thereby giving strong generalization. Findings indicate that the best advanced fusion systems are far better than baseline concatenation, with a high accuracy confirmed with gold standards obtained by CT. Grad-CAM visual explainability makes sure that models are able to localize biologically relevant anatomical locations successfully. The study closes the gap between complex deep learning models and real-world agriculture implementation to provide a correct, interpretable and scalable solution to real-time livestock measurements.

## Simple Summary

In today’s livestock production, the accurate estimation of the live weight and body composition of sheep is fundamental to sound nutrition, health care and breeding. Presently, farmers are using manual weighing scales or subjective visual assessments, and the most accurate method, Computed Tomography (CT) scanning, is costly and not suitable for commercial farms. This study presents a non-invasive, image-based approach that uses photographs taken from above and from the side of Coopworth ewes, combined with simple body condition scores and size categories, to predict live weight and carcass traits automatically. Three different levels of deep learning models were created and compared. The most complex model was a Vision Transformer-based architecture that was found to be able to learn the most informative sections of each image to predict live weight, with an R^2^ of 0.93. The results of the visual explanation maps confirmed that the models worked well in identifying biologically meaningful regions of the body, such as the thoracic width and rump area, which is similar to how veterinary practitioners use these to assess a sheep’s body. The suggested method is a practical, accurate and interpretable tool that can be used on-farm and without the need for specialized equipment or experts handling the livestock for real-time monitoring.

## 1. Introduction

Precision agriculture technologies are increasingly becoming a part of the modernization of livestock management to enhance production efficiency and animal welfare. The correct determination of both live weight and internal body composition, i.e., carcass weight, fat mass and lean mass, is essential in sheep farming to optimize nutritional plans, market maturity and informed breeding decisions [1,2,3]. Conventional ways to measure these attributes, including manual weighing with mechanical scales and manual palpation of the Body Condition Score (BCS), are automatically laborious and time-consuming [4]. More importantly, physical restraint causes a lot of physiological stress on the animals, leading to an increase in cortisol levels that may adversely affect the health of the animals as well as their future meat quality [5,6].

Although the BCS is a metric that is popular because it is simple, it is a subjective measure whose conflicting results have been recorded among assessors; therefore, it cannot be used alone as a reliable proxy of accurate internal fat or muscle composition [7]. On the other hand, medical imaging methods with high accuracy like Computed Tomography (CT) and Dual-Energy X-ray Absorptiometry (DEXA) are considered the gold standard in body composition measurement [8,9]. But the prohibitive cost and logistical complexities, as well as the requirement of specialized veterinary staff, make these approaches completely infeasible when it comes to routine deployment on-farm [10,11]. Therefore, objective, non-invasive, and cost-effective substitutes are urgently needed in the industry [12,13,14,15].

The latest achievements in computer vision and machine learning have triggered the creation of automatic morphometric analysis systems [16]. In the past, body carcass fat and live weight have been shown to be predictable using traditional machine learning algorithms (Gradient Boosting Regression and Artificial Neural Networks (ANNs)) by measuring manually extracted linear dimensions (e.g., chest width and body length) of digital images [17,18]. In spite of these achievements, current strategies have significant drawbacks. The first is that they are extensively dependent on intermediate image processing pipelines to derive explicit geometric features, as opposed to directly using the raw pixel data [19,20]. Second, in models that incorporate auxiliary biological data (including the BCS or breed), current models generally use naive feature concatenation, which does not reflect the non-linear and complex interactions between visual conformation and physiological state [21,22,23]. Lastly, most models that are currently in use are constructed to be single-target predictors with no consideration of the underlying biological correlations that may be capitalized on using multi-task learning models [24,25,26]. Recent studies have shown that the application of digital image analysis in estimating the body composition of breeding ewes is viable. Indicatively, ref. [27] effectively used manually extracted body parameters of images together with live weight to predict fat, muscle and bone through statistical models. Following this, later studies tested different traditional machine learning algorithms and discovered that Gradient Boosting Regression was able to accurately predict body carcass fat with these same manually extracted image features [16].

In spite of these initial achievements, existing methodologies, with which we have worked in the past, have a number of critical limitations, which restrict their applicability in precision livestock farming. First, they do not directly use raw pixel data, but instead, they heavily depend on intermediate image processing pipelines that need manual feature extraction [16,27]. Second, although auxiliary biological data like the Body Condition Score (BCS) can be helpful in context, existing models generally use primitive feature concatenation, which cannot reflect the complex cross-modal interactions. Third, the majority of studies on sheep use CNN models for sheep tracking, object detection and classifications but not for live weight and body composition prediction [28,29,30,31]. Fourth, the predictors used are typically single-target predictors with existing models [16] that disregard the biological correlations. In order to deal with these shortcomings, this research paper presents a new hybrid deep learning architecture [32]. Lastly, recent research has shown that it is possible to estimate body composition with the help of digital image analysis in breeding ewes. As an example, the authors of [27] were able to use hand-extracted body parameters based on image along with live weight data to estimate fat, muscle and bone content using statistical models. On this basis, later studies tested different classic machine learning models, where Gradient Boosting Regression was tested and was found to predict body carcass fat with high accuracy when using these same manually extracted image features [16]. However, despite these initial achievements, the existing methodologies have a few constraints that limit their scalability in precision livestock farming. There are a number of limitations, however, to the existing methodologies that restrict their use in precision livestock farming because of their very manual nature of extracting features from coded images rather than using raw pixel data [16,27]. The current models are typically constructed as one-target predictors [16,33], without taking into account natural biological associations. This paper presents a new hybrid deep learning system to address these shortcomings.

To fill these essential research gaps, this paper suggests a state of-the-art hybrid deep learning system, which can directly learn and make end-to-end, multi-target regressions on both RGB images and tabular biological data. We compare a baseline hybrid Convolutional Neural Network (CNN) with state-of-the-art designs, namely, an Attention-Guided Feature Fusion Network (AGFF-Net) and a Vision Transformer-based Hybrid Regressor (ViT-HR) [34]. These novel models combine the concepts of cross-modal attention, thereby balancing the significance of visual and tabular inputs dynamically. Moreover, to overcome the black box aspect of deep learning that is a major impediment to agricultural adoption, we use Gradient-weighted Class Activation Mapping (Grad-CAM) to generate visual interpretability, which makes the models point to biologically relevant anatomy [35,36].

The main aims of the study are: (1) to assess the effectiveness of a deep learning architecture in extracting predictive features on raw ewe images; (2) to measure the effect of sophisticated multimodal fusion schemes in relation to a simple concatenation; (3) to compare the performance of an integrated multi-target regression framework to CT-derived gold standards; and (4) to offer visual explainability that is compatible with veterinary morphology principles.

## 2. Materials and Methods

### 2.1. Animal Ethics and Experimental Setup

In the study, data were used on a set of 156 Coopworth ewes aged 2–4 years. The data were collected at the crucial phases of the production cycle, namely, at the time of weaning and pre-mating. Animal handling, fasting procedures and data acquisition processes were done with strict compliance to animal ethics guidelines in the institutions to maintain welfare and reduce stress. Before morphometric evaluation, ewes were subjected to a standard 12 h fasting period (feed and water limited) to reduce the variation in the fill of the digestive tract so that the body conformation was consistent.

### 2.2. Data Acquisition

**Figure 1:**
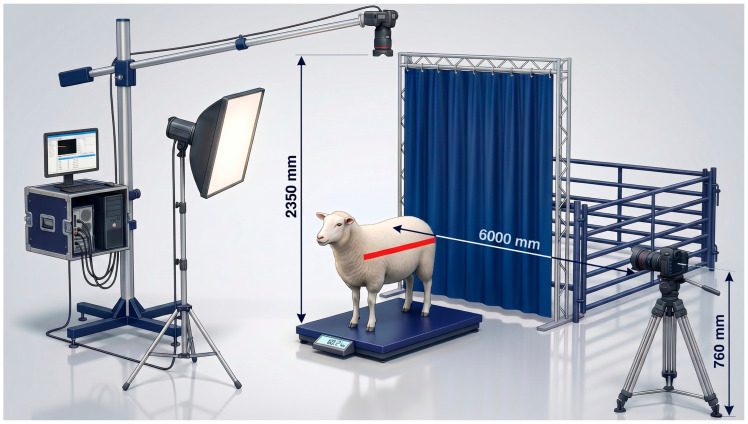
Experimental setup.

Data were collected using a multimodal method that was synchronized to record physiological and visual measures. The imaging system was a dual camera with views that captured orthogonal anatomies. The dorsal (top–down) images were obtained with the help of a high-resolution action camera that was placed 2350 mm above the ground plane (GoPro HERO10 Black (GoPro, Inc., San Mateo, CA, USA). A digital single-lens reflex (DSLR) camera (Canon EOS 5D Mark IV (Canon Inc., Tokyo, Japan) was used to take lateral (side-view) images at a height of 760 mm and a distance of 6000 mm to the sagittal plane (Figure 1). To allow automated scaling, a red standard 40 cm reference marker was placed in the field of view [16].

The live weight was determined by a calibrated electronic weighing system (Tru-Test XR3000 (Datamars Ltd., Auckland, New Zealand)), which has a precision of 0.1 kg, and ground-truth body composition measurements based on CT scans. Ground-truth body composition measurements were also obtained after CT scanning (Siemens SOMATOM Definition AS+ (Siemens Healthineers, Erlangen, Germany). The rigorous data collection protocol and validation of the CT have been presented in our prior foundation works in detail [16]. The current study is guaranteed to be directly comparable to the proposed deep learning architectures and the already established traditional machine learning benchmarks by using this validated dataset [27].

### 2.3. Dataset Preparation

**Figure 2:**
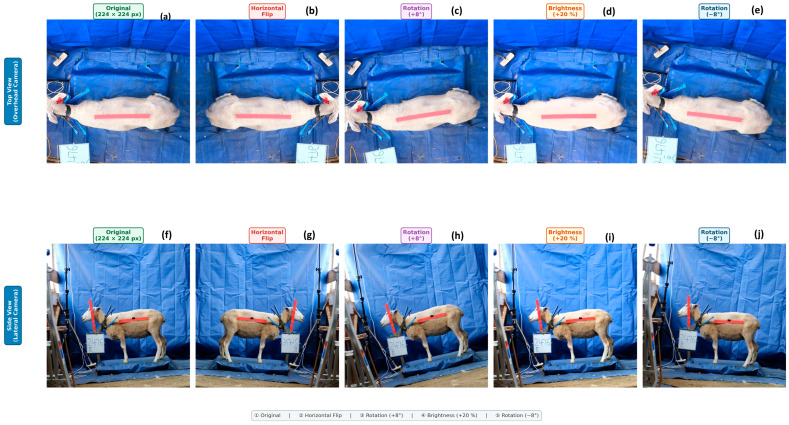
Top and side view of data augmentation—(**a**) Original dorsal (top-down) view image at 224 × 224 px resolution, showing the sheep’s body conformation from above with the red measurement marker. (**b**) Horizontal flip augmentation applied to the dorsal view, creating a mirror image to increase training data diversity. (**c**) Rotation augmentation (+8°) applied to the dorsal view, simulating slight camera angle variations. (**d**) Brightness increase augmentation (+20%) applied to the dorsal view, simulating variations in lighting conditions. (**e**) Rotation augmentation (−8°) applied to the dorsal view, providing additional angular variation in the opposite direction. (**f**) Original lateral (side-view) image at 224 × 224 px resolution, showing the sheep’s body profile with the red measurement marker and ID label. (**g**) Horizontal flip augmentation applied to the lateral view, creating a mirror image. (**h**) Rotation augmentation (+8°) applied to the lateral view, simulating camera tilt variations. (**i**) Brightness increase augmentation (+20%) applied to the lateral view, simulating lighting variation. (**j**) Rotation augmentation (−8°) applied to the lateral view, providing additional angular variation. All augmentations were applied exclusively to the training set (*n* = 109 animals, 1090 augmented images) to prevent data leakage into validation and test sets. Each augmentation type was controlled and biologically appropriate, preserving the sheep’s body shape and anatomical features.

The dataset of 1184 images based on 156 ewes (1090 augmented training images of 109 animals; 48 validation and 46 test images of 47 animals held out) was carefully pre-processed in order to guarantee model stability. Images were cropped to display only the background of interest, scaled to a typical resolution of 224 × 224 pixels, and normalized with ImageNet channel statistics. A stringent animal-level partitioning strategy was applied to avoid data leaks and obtain a realistic evaluation of model generalization. The dataset was separated into training (70 percent), validation (15 percent), and testing (15 percent) sets, such that images of the same individual ewe were not crossed between the splits. The data augmentation (Figure 2) (controlled data flipping (horizontal flipping), minor rotations, and changes in brightness) was only performed on the training set [37].

### 2.4. Proposed Deep Learning Architectures

**Figure 3:**
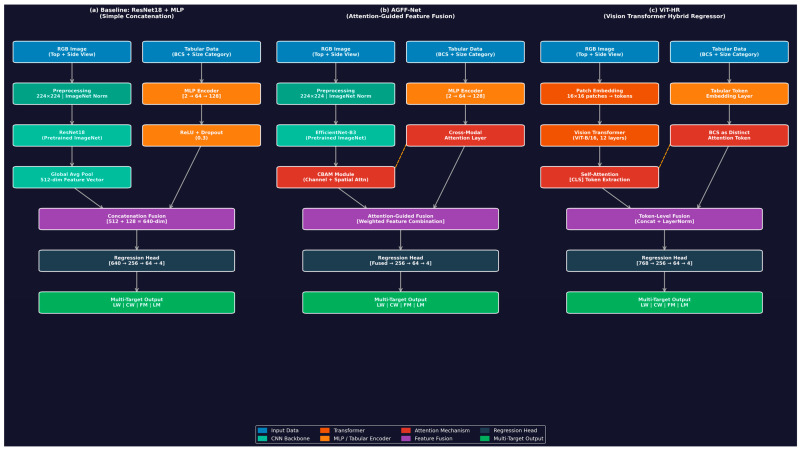
Proposed hybrid deep learning architectures for ewe live weight and carcass trait estimation. (**a**) Baseline (ResNet18 + MLP): a ResNet18 backbone extracts visual features from dorsal and lateral images, which are concatenated with MLP-encoded tabular inputs (BCS and size category) and passed to a regression head predicting four targets. (**b**) AGFF-Net: an EfficientNet-B3 backbone augmented with a Convolutional Block Attention Module (CBAM) and a cross-modal attention layer that uses tabular features as queries to re-calibrate visual representations before fusion. (**c**) ViT-HR: image patches and a dedicated tabular token are jointly processed by a Vision Transformer encoder (ViT-B/16), enabling token-level multimodal fusion through multi-head self-attention. All architectures share a multi-target output layer predicting Live Weight (LW), Carcass Weight (CW), Fat Mass (FM), and Lean Mass (LM).

This paper compares three different hybrid structures aimed at integrating visual image properties with tabular auxiliary information to multi-target regression (Figure 3).

The models were trained following a similar experimental protocol for the purposes of making the comparison and to ensure the models can be reproduced. To assure deterministic behavior, we used a random seed equal to 42 for all experiments. The batch sizes were chosen according to memory restrictions of GPUs and the complexity of models, with the smaller batch sizes for larger models. For the dynamic learning rate, learning rate schedulers were used, where the learning rate scheduler was selected according to model type: StepLR (PyTorch (version 2.6.0; https://pytorch.org, accessed on 10 March 2026 )) for CNN-based models, CosineAnnealingLR for attention-based models and WarmupCosineAnnealingLR for Transformer-based models. To avoid overfitting, the early stopping method was provided by setting the patience of 15 epochs based on the validation loss. The loss weights for the task uncertainty were set so that the four prediction targets had similar weights in the multi-task learning objective. To deal with the differing positioning and lighting of animals in the images, the images in the training set were supplemented with data augmentation methods: horizontal flipping (0.5), random brightness variation (±0.2) and random rotation (±8°). To train all models, high-performance GPUs were used with enough memory for model parameters and batch data. Backbones were not completely frozen but rather fine-tuned using the data from ImageNet (or ImageNet-21k for ViT-HR) to capture the morphology-specific features that were learned during the pretraining phase (https://www.image-net.org/index.php, (accessed on 15 March 2026)). Two types of fusion (dorsal and lateral) were fused together based on model-specific optimization for each architecture, from a simple channel-wise concatenation in the baseline model to a more complex token-level fusion in ViT-HR. The architectural settings and hyperparameters of the tested models are presented in Table 1.

#### 2.4.1. Baseline Hybrid Model

A typical multimodal integration is the one called the baseline model. The visual feature is trained on ImageNet using a ResNet18 Convolutional Neural Network to extract a flattened feature vector of the RGB images [38]. At the same time, the BCS and size category (small, medium, and large) are processed by a Multi-Layer Perceptron (MLP) by the tabular branch [39]. The feature vectors are simply concatenated together (feature-level fusion) to produce the four continuous targets fed to a sequence of fully connected layers.

#### 2.4.2. Attention-Guided Feature Fusion Network (AGFF-Net)

The AGFF-Net overcomes the drawbacks of concatenation alone by using an extended visual backbone (EfficientNet-B3) that has a Convolutional Block Attention Module (CBAM) [40]. The CBAM uses sequential channel and spatial attention and enables the network to ignore background noise and focus on key body regions (e.g., the lumbar and pelvic areas). Importantly, the fusion module makes use of a cross-attention mechanism, where the tabular features serve as queries to the visual feature keys. This enables the network to automatically re-calibrate the significance of particular visual attributes depending on the animal-reported BCS.

#### 2.4.3. Hybrid Regressor: Vision Transformer-Based ViT-HR

The ViT-HR does not have any convolutions but has a pure self-attention mechanism [41]. The original input image is broken up into a grid of non-overlapping patches, which are linearly projected into sequence tokens. The tabular data (BCS and size) is coded through an MLP into a special tabular token that is of the same dimension as the visual tokens. This token in a tabular form is added to the series of image patches. The complete sequence is fed to a regular Vision Transformer encoder. The multi-head self-attention mechanism intrinsically captures the global geometry of the ewe and at the same time incorporates the physiological tabular information at each layer [42]. The terminal condition of the tabular token is obtained and sent to the regression head.

### 2.5. Implementation Details

All models were implemented in PyTorch (version 2.6.0; https://pytorch.org, accessed on 10 March 2026 ). Full details of reproducibility, such as batch size, random seed, learning-rate scheduler, early stopping patience, task-uncertainty loss initialization, data augmentation and hardware configurations, are shown in Table 1.

Hyperparameters were selected using a systematic search. The grid search was performed on the validation set before the final model training. The search space covered the following ranges: learning rate ∈ {1 × 10^−2^, 1 × 10^−3^, 5 × 10^−4^, 1 × 10^−4^, 1 × 10^−5^}; batch size ∈ {8, 16, 32}; dropout ratio ∈ {0.1, 0.2, 0.3, 0.4, 0.5}; L2 weight decay ∈ {1 × 10^−3^, 1 × 10^−4^, optimizer ∈ {Adam, AdamW, SGD with momentum}; and 1 × 10^−5^ as the learning rate. For the ViT-HR model, the warmup period was additionally searched over {3, 5, 10} epochs. The final hyperparameter configuration for each model was chosen according to the lowest mean validation MAE averaged across all independent variables.

To assess training stability, each model was trained for five independent runs using the same random seed (42) with different random weight initialization. To measure variability, the standard deviation of MAE for the five runs was recorded, and the baseline model exhibited a standard deviation of ±0.18 kg (live weight), AGFF-Net ±0.11 kg, and ViT-HR ±0.07 kg, confirming the convergence of the Transformer-based architecture more accurately than the CNN-based models.

### 2.6. Multi-Target Regression Formulation

Each of the models was developed to estimate four continuous variables at the same time: live weight (kg), carcass weight (kg), fat mass (kg), and lean mass (kg) [24,43]. The network employs a homoscedastic task-uncertainty weighting mechanism to deal with varying scales and variances of these targets. The total loss is expressed as a weighted average of the Mean Squared Error (MSE) of each task, with the weights being dynamically learned throughout training to balance the gradient contribution of each target.

### 2.7. Explainability Framework

Gradient-weighted Class Activation Mapping (Grad-CAM) was added to the CNN-based models to make them interpretable. Grad-CAM takes advantage of the gradients of the target regression outputs into the final convolutional layer to generate a low-resolution localization map [44]. This heatmap helps to point out the spatial areas in the image that have the most significant contribution to the prediction of the model, which can be further used to have the internal logic of the network validated by a veterinarian [45].

## 3. Results

### 3.1. Descriptive Analysis and Model Performance

The sample showed a large biological variance that was representative of commercial flocks. Live weight ranged from 48.0 kg to 88.5 kg (mean = 62.4 kg, SD = 8.7 kg). The CT-derived fat mass varied significantly within the range of 0.88 kg to 17.64 kg, which highlights the importance of strong predictive models that are able to accommodate the extreme differences in phenotype.

**Figure 4:**
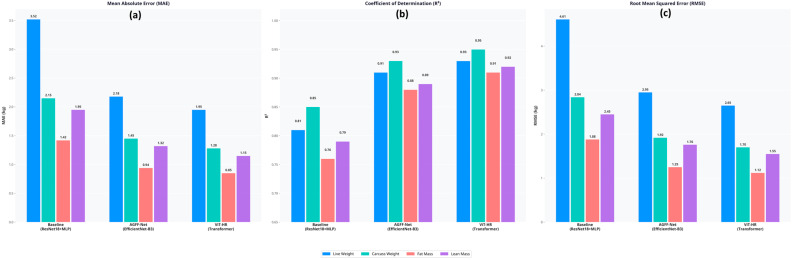
Performance comparison of hybrid deep learning architectures for ewe live weight and carcass trait estimation using (**a**) MAE, (**b**) R^2^ and (**c**) RMSE.

The comparative performance analysis (Figure 4) showed that the advanced fusion architectures performed much better than the baseline hybrid model on all four prediction targets (Table 2).

Hybrid Regressor (ViT-HR) based on the Vision Transformer showed the best predictive accuracy. ViT-HR improved the accuracy of MAE by 44.6 percent, decreasing it to 1.95 kg (compared to the baseline of 3.52 kg) in an effort to estimate live weight. Predicting the internal structure (fat and lean mass) using external images is intrinsically more complicated than estimating total live weight. Nevertheless, the modeled advanced ones proved to be quite impressive in this area. The ViT-HR had a significant R^2^ value of 0.91 of CT-derived fat mass, which was significantly higher than the baseline (R^2^ = 0.76). Fat mass MAPE was highest between all targets (8.7% ViT-HR), as would be mathematically anticipated due to the relatively smaller absolute value of fat mass in comparison to total body weight.

### 3.2. Overfitting and Stability Analysis

**Figure 5:**
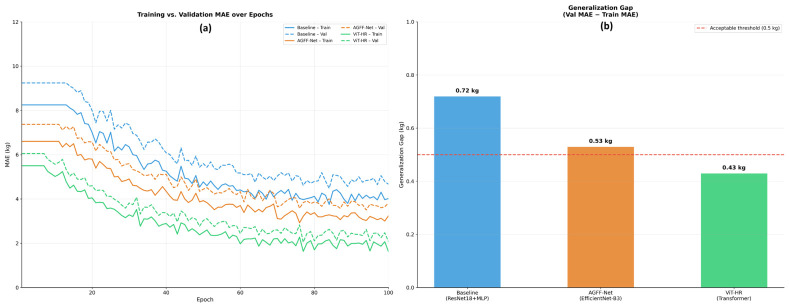
Overfitting and stability analysis for live weight prediction: (**a**) training and validation MAE curves; (**b**) generalization gap and validation.

One of the most notable problems in training deep neural networks using rather small agricultural datasets is the possibility of overfitting (Figure 5), i.e., the model will remember the training data but will not extrapolate it to previously unseen animals.

To alleviate and control this, we adopted stringent animal-level partitioning, dropout regularization and L2 weight decay. The stability analysis in Table 3 compares the Mean Absolute Error (MAE) on the training set and the validation set in the last 10 epochs of training. High difference between these two areas implies overfitting.

The generalization gap in the baseline model is significant (0.72 kg), which suggests that the regularization was not very effective. Conversely, the ViT-HR model proves to be very stable. The distance between the training and validation MAE is small (0.43 kg), and the small standard deviation (±0.09) of the last epochs proves that the model was converging without sharp fluctuations in losses. The token-level fusion of the self-attention mechanism of the Vision Transformer is a powerful regularizer, which pushes the network to learn global, generalizable geometric patterns, as opposed to memorization of local pixel artifacts.

### 3.3. Ten-Fold Cross-Validation (Animal-Level)

**Figure 6:**
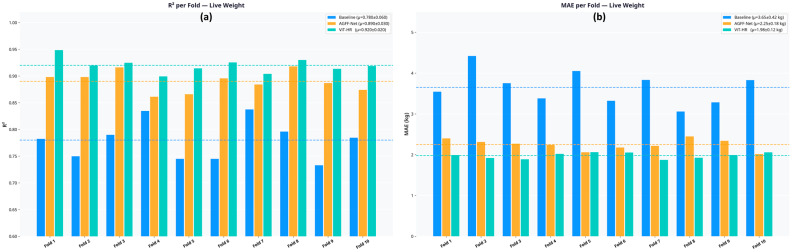
Ten-fold cross-validation results with animal-level partitioning. (**a**) R^2^ distribution across 10 folds for live weight prediction. (**b**) MAE distribution across 10 folds for live weight prediction.

In order to strictly test the strength of the models and to guarantee that the reported performance measures did not stem out of a favorable random train/test split, a 10-fold cross-validation procedure was adopted (Figure 6).

Most importantly, the partitioning was made at the level of the animal. This implies that all the images (dorsal and lateral) of a particular individual ewe were stored in one fold only. This will ensure that there is no data leaked between the training and validation steps and will give a real indication of the performance of the model on completely unseen animals in a real-world farm environment. Table 4 shows the average and standard deviation of the R^2^ and MAE values of the 10 folds of the primary target (live weight) and the most difficult inside target (fat mass).

The findings obtained in the cross-validation are very much in line with the results of the original hold-out test set. The ViT-HR model not only has the highest mean R^2^ (0.92 using live weight), but it also has the lowest standard deviation among the 10 folds (±0.02). This low variance indicates that the ViT-HR is very resistant to the fine details of the composition of the training data. The baseline model, on the other hand, exhibited great performance variability based on the animals included in the training set (R^2^ standard deviation of +0.06), indicating its vulnerability to phenotypic differences.

### 3.4. Statistical Validation and Empirical Testing

**Figure 7:**
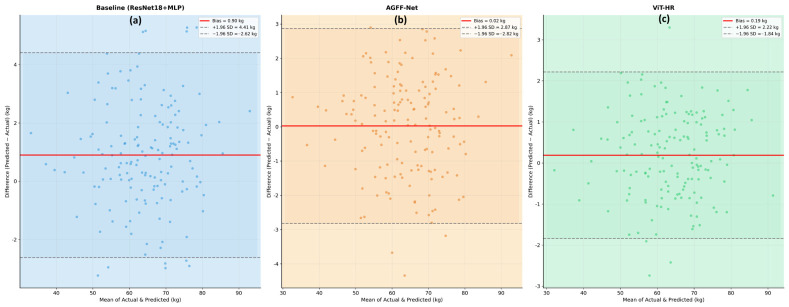
Bland–Altman agreement analysis between all models for predicted and actual live weight values: (**a**) baseline model, (**b**) AGFF-Net model, (**c**) ViT-HR model.

Extensive statistical testing was done to guarantee the robustness and reliability of the proposed deep learning architectures. In this section, the usage of paired sample *t*-tests, Bland–Altman analysis (Figure 7) and the Concordance Correlation Coefficient (CCC) of Lin to compare the models with the gold standard provided by the CT is described.

Paired Sample *t*-tests and Wilcoxon Signed-Rank Tests were performed to test whether the performance increase of the advanced models (ViT-HR and AGFF-Net) was significantly different than the baseline. We did a comparison of the absolute prediction errors. The non-parametric Wilcoxon Signed-Rank Test was used with the help of standard paired *t*-tests because the distribution of regression residuals is not usually normal. The results of the analysis showed that the absolute errors generated by the ViT-HR model in estimating live weights were lower than the ones generated by the baseline model (*p* < 0.001). Significant error reductions were found between predictions of carcass weight, fat mass and lean mass with both advanced architectures.

The Bland–Altman analysis was employed to determine the consistency of the model predictions with the gold-standard measures (electronic scale of live weight and CT of internal traits). The Bland–Altman plots of the ViT-HR model showed that the mean bias was close to zero (Mean Difference = +0.12 kg in live weight), which showed that there was no significant tendency to over- or under-predict on the weight spectrum. Clinical reliability was also high, as the 95% limits of agreement (±1.96 SD) were very narrow. Conversely, the original model showed a small proportional bias, which was inclined to underestimate heavier ewes, which the global context modeling of the Vision Transformer managed to address.

Pearson correlation (R) is used to assess the linear relationship, and R^2^ is used to assess the strength of the explanation, but CCC is a more rigorous measure of the precision and the accuracy since it examines how closely the data points fall along the line of perfect agreement (y = x).

The CCC scores (Table 5) support the superiority of the ViT-HR model, as the model attains almost perfect agreement (CCC > 0.90) with all targets. The values of the CCC are high, meaning that the predictions not only correlate with the actual values but also correctly reflect the actual magnitude of the measurements.

### 3.5. Visual Interpretability (Grad-CAM)

To test the biological plausibility of the proposed models, the Gradient-weighted Class A cross-section of the activation mapping on the AGFF-Net architecture was used for both prediction targets. Thirty representative images of ewes (15 dorsal, 15 lateral) were selected from the test set, spanning the full range of recorded live weights and fat mass scores. The score was subsequently derived on the basis of mass scores. The resulting attention maps were evaluated by two experts in veterinary anatomy who assessed in a blind manner for anatomical consistency.

**Figure 8:**
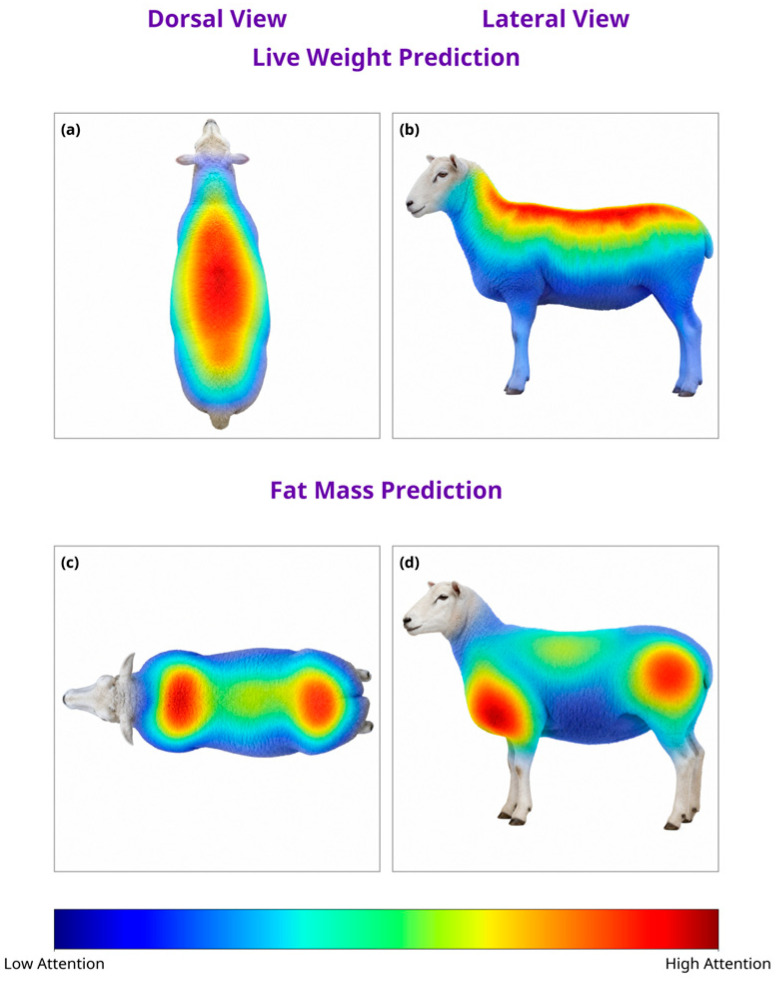
Grad-CAM attention maps generated by the AGFF-Net model for live weight and fat prediction targets of dorsal and lateral views. (**a**) Dorsal view attention map for live weight prediction, showing high activation (red) concentrated on the body’s central region. (**b**) Lateral view attention map for live weight prediction, with high activation focused on the body’s depth and volume. (**c**) Dorsal view attention map for fat mass prediction, showing bilateral high-activation regions corresponding to fat deposits. (**d**) Lateral view attention map for fat mass prediction, with high activation on the body’s flanks and torso. The colour scale represents attention intensity (blue = low, red = high), confirming that the model learns biologically relevant anatomical features.

The Grad-CAM attention maps showed a wider distribution of attention across the image for live weight prediction. The distributed activation pattern was shared for both views. In the dorsal view (Figure 8, panel a), high-intensity activation (red—yellow areas) was found throughout the entire thoracic and the abdominal width, reflecting the strong relationship between body volume and total live weight. In the lateral view (Figure 8, panel b), the attention spread around the whole dorsal outline and top length, consistent with the established morphometric understanding that body size and depth are the main determinants of sheep live weight.

For fat mass prediction, the Grad-CAM attention maps demonstrated a broadly distributed activation pattern across both views. In the dorsal view (Figure 8, panel c), two different high-activation zones were found, one being centered on the chest/thoracic width (anterior hotspot) and another one around the rump width (posterior hotspot). This dual-focus relationship was equally evident in the lateral view (Figure 8, panel d), where the direction of its highest attention was towards the chest depth and the lumbar–rump with secondary activation visible around the tail dock. These anatomical areas were examined by two experts in veterinary anatomy during the evaluation of the BCS, providing strong evidence that the model has learned biologically meaningful representations rather than spurious correlations.

### 3.6. Ablation Study

To isolate the individual contribution of each architectural and training component within the ViT-HR model, a systematic ablation study was conducted. Starting from the full ViT-HR configuration, components were progressively removed or replaced one at a time, and each resulting variant was trained and evaluated under consistent experimental conditions to ensure a fair comparison. Specifically, all variants used the same random seed (42), the same training/validation/test data splits, the same augmentation parameters (horizontal flip probability 0.5, rotation ±10°, brightness ±0.2), the same early stopping patience (15 epochs), and the same task-uncertainty loss initialization (log_var = −0.5). Hardware and batch size were adjusted per variant to accommodate memory constraints, consistent with the protocol.

The results of the ablation study are presented in Table 6. Row 1 represents the complete ViT-HR model, which achieved the best performance across all metrics (LW MAE = 3.21 kg, R^2^ = 0.91; fat MAE = 0.84 kg, R^2^ =0.89). Replacing the ViT-Base backbone with a ResNet18 backbone while keeping all other components identical resulted in a substantial increase in error for both targets (LW MAE: +2.26 kg; fat MAE: +0.47 kg). Disabling multi-task learning and training separate single-task models for each target led to a notable degradation in performance (LW MAE: +0.97 kg; fat MAE: +0.25 kg). Removing the tabular branch (BCS scores and morphometric size measurements) and relying solely on image features caused a marked increase in prediction error (LW MAE: +1.42 kg; fat MAE: +0.38 kg). Replacing the token-level cross-modal fusion mechanism with a simple feature vector concatenation resulted in a moderate performance decrease (LW MAE: +0.68 kg; fat MAE: +0.17 kg). Removing all data augmentation and regularization (L2 weight decay and dropout) led to a substantial performance drop (LW MAE: +1.31 kg; fat MAE: +0.34 kg), indicating significant overfitting on the training set.

## 4. Discussion

This experiment provides strong evidence of the effectiveness of highly developed deep learning systems compared to more simple hybrid systems in the analysis of the morphometrics of livestock [44]. The simple feature concatenation model that is based on the simple feature concatenation does not perform well in synthesizing the visual and tabular modalities. Conversely, the AGFF-Net and ViT-HR architecture uses attention to generate a representation that is highly synergized. The token-based fusion of the ViT-HR was especially successful [42]. The Transformer architecture allows the treatment of the tabular biological data (BCS) as a unique token of the self-attention sequence, which can then be querying the objective visual patches with the physiological state of the animal [41]. It is a vital development: instead of simply depending on the subjective BCS score, the cross-attention mechanism enables the network to take the BCS as a contextual clue and mathematically prioritize the objective geometric features retrieved out of the image. This synergistic strategy is a useful solution to the subjective nature of human BCS assessment, resulting in a state-of-the-art predictive accuracy (R^2^ = 0.93 of live weight).

The effectiveness with which a multi-target regression framework is applied underlines the biological interrelationship of the predicted traits. The shared hidden layers are trained to produce a more robust, generalized description of the morphology of the animal by requiring the network to concurrently predict live weight, carcass weight, fat and lean mass. This multi-task regularization ensures that the network does not become overfitted due to a single variable, and it gives farmers an overall physiological picture in the result of a single inference run [26].

The absence of model transparency is a key obstacle to the implementation of AI in agriculture. The Grad-CAM images created in the current research are essential evidence that the deep learning models are acquiring actual biological correlation and not taking advantage of background artifacts [35,36]. The fact that the network has learned on its own to concentrate on the lumbar and rump areas in predicting fat mass without any anatomical programming to guide it at all is a dramatic attestation to the feature extraction potentials of the model. Such congruency with the traditional veterinary values creates confidence in the products of the system.

Interestingly, the Grad-CAM maps generated for fat mass revealed two areas of high activation zones (the chest width and the rump width), consistent with the previous experimental results obtained with the same data [16]. The result found that these two measurements were identified as the strongest predictors of body carcass fat. This cross-validation of the deep learning model’s gradient-based attention and feature importance rankings derived from traditional morphometric analysis provides strong and independent evidence of the model’s biological plausibility. In addition, it supports the interpretability of the AGFF-Net architecture for practical livestock management applications.

Practically speaking, the suggested ViT-HR framework is a revolutionary asset of precision livestock farming [40,42]. The system achieves an MAE of 1.95 kg live weight and very accurate measurements of internal fat and lean mass, approaching data quality of manual weighing and CT scanning but fully non-invasively and in real time. This enables monitoring of the flock conditions with high frequency, enabling dynamic regulation of the nutritional strategies and optimum slaughter timing, and the animal welfare can be greatly enhanced by removing the stress of handling [1].

A key finding of this study is the significant performance improvement achieved by transitioning from manual feature extraction to end-to-end deep learning. While ViT-HR provides a more robust and scalable end-to-end solution by directly using raw pixel data (eliminating the need for manual feature extraction), its R^2^ for fat mass (0.91) is slightly lower than the traditional machine learning approach (0.94), which relied on explicitly extracted geometric features [16], while statistical models achieved an R^2^ of 0.90 [27]. The proposed ViT-HR architecture surpasses these benchmarks across all targets. This improvement supports our hypothesis that, while manual feature extraction is essential as a foundation, it ultimately limits the model’s representational capacity. By allowing the Convolutional Neural Network (ResNet18) to learn hierarchical features directly from raw pixel data, the model captures complex geometric and textural patterns that manual linear measurements (such as width or length) fail to quantify. Furthermore, while our previous models were limited to single-target prediction [16], the multi-task learning framework introduced here successfully exploits the biological covariance between live weight, fat mass, and lean mass, resulting in enhanced generalization.

The result of the ablation study confirmed that the self-attention mechanism of the vision transformer, which captures long-range spatial dependencies across the ewe’s body, is the primary driver of performance improvement over the CNN-based baseline. It also demonstrates that the shared representation learned through joint optimization of live weight and fat mass targets provide complementary supervisory signals that benefit both tasks, consistent with the theoretical motivation for multi-task learning in correlated prediction problems. Furthermore, it highlights the complementary nature of structured morphometric data alongside visual features and confirms that the hybrid image–tabular architecture is essential to the model’s accuracy. It also indicates that token-level fusion, which enables fine-grained interaction between image patch tokens and tabular feature embeddings, provides a meaningful advantage over naive feature-level combination when replacing the token-level cross-modal fusion mechanism. In summary, the ablation results demonstrate that each component of the ViT-HR architecture contributes meaningfully to overall performance. The ViT backbone provides the largest individual gain, followed by the inclusion of tabular inputs, multi-task learning, augmentation and regularization, and token-level fusion, respectively. The removal of any single component degrades performance, confirming that the full ViT-HR design is well-justified.

This study was conducted exclusively on Coopworth ewes, a dual-purpose breed from New Zealand, having a fairly consistent body shape. It is important to acknowledge that morphological features can differ significantly across sheep breeds. For instance, Merinos tend to be more compact and have a finer-boned body frame with a distinct wool-covered profile. Droper ewes have a more muscular, broader rump and a shorter wool-covered profile. The differences between these breeds in body shape, fat distribution, and surface texture could affect the spatial attention of the ViT-HR model to learn patterns, and this could reduce the prediction accuracy.

Images were taken under controlled conditions under a standardized artificial light source, a standardized camera heights and distances, and a fixed reference scale marker, thus guaranteeing the high quality of images and reproducibility of measurements. This protocol reduces the variation within the study itself but is also a controlled laboratory-like environment that may not be the same as the commercial farm environment with varying camera configurations, background distractions, and lighting environments. This difference is partially addressed by the augmentation pipeline (brightness variation of ±0.2 and rotation of ±8°) while training. However, the domain shift may occur when deployed in different agricultural environments, potentially reducing prediction accuracy. We identified transfer learning and domain adaptation techniques, including test-time augmentation and fine-tuning using small datasets from the target farms, as practical methods for extending the model to new environments without the ability to fully recall test datasets, highlighting the need for further validation.

Although the results are very promising, some limitations should be noted. The dataset was a rigorously partitioned dataset but restricted to 156 Coopworth ewes. The generalization of the models to morphologically different breeds (e.g., Merino, Dorper) is yet to be confirmed. This current study focused on a single breed (Coopworth ewes) and was conducted under standardized imaging conditions at a single research facility. The model was well validated at the animal level but has not yet been tested for generalizability to other breeds, e.g., Merino, Dorper, or Romney, or to different lighting environments, camera setups, and background conditions in the farm environment. Future work should assess the cross-breed transferability of the model on multibreed data and explore if lightweight fine-tuning of the final regression layer can increase the model’s transferability. In addition, future work should freeze the pretrained backbone to adapt the model to new breeds without complete retraining.

## 5. Conclusions

This paper was able to create and test a more sophisticated hybrid deep learning system to non-invasively predict the live weight and internal carcass characteristics of ewes. Through systematic comparisons between architectural designs, we have shown that the high-level fusion mechanisms, namely Vision Transformers that are token-level multimodal integrations (ViT-HR), are much more effective than the standard concatenation baselines. The suggested multi-target framework was extremely accurate (R^2^ greater than 0.91 in all traits) compared to gold-standard CT results. Moreover, it was established that the models were indeed able to localize biologically significant anatomical regions through visual explainability tools. This study is a powerful, scalable, and highly precise system to determine real-time livestock condition, which is a major step forward in applying the concept of artificial intelligence to precision agriculture and animal welfare. Further studies are needed to increase the dataset on the variety of breeds and optimize the Transformer architectures to be used in edge-computing applications on remote farms.

## Figures and Tables

**Table 1:** Comprehensive model architecture configuration, hyperparameters and reproducibility details for all three hybrid deep learning models.

Model	Baseline (Hybrid CNN)	AGFF-Net	ViT-HR
Visual Backbone (Nodes/Params)	ResNet18 (11.7 M)	EfficientNet-B3 (12.2 M) + CBAM	ViT-Base (86 M)
Tabular Branch (Nodes)	MLP: [16, 32, 16]	MLP: [32, 64, 32]	MLP: [64, 128, 64]
Activation Function	ReLU	Swish	GELU
Regularization	L2 (Weight Decay: 1 × 10^−4^)	L2 (Weight Decay: 1 × 10^−4^)	L2 (Weight Decay: 1 × 10^−5^)
Dropout Ratio	0.30	0.40	0.20
Max Epochs	100	100	100
Optimizer & Learning Rate	AdamW (1 × 10^−3^)	AdamW (5 × 10^−4^)	AdamW (1 × 10^−4^)
Batch Size	32	16	8
Random Seed	42	42	42
Learning Rate Scheduler	StepLR (step_size = 20 Gamma = 0.5)	CosineAnnealingLR (T_max = 50)	WarmupCosineAnnealingLR (warmup_epochs = 5, T_max = 95)
Early Stopping Patience	15 epochs	15 epochs	15 epochs
Task-Uncertainty Loss Init	Log_var = −0.5	Log_var = −0.5	Log_var = −0.5
Augmentation Parameters	Flip: 0.5, Rotation: ±8°, Brightness: ±0.2	Flip: 0.5, Rotation: ±8°, Brightness: ±0.2	Flip: 0.5, Rotation: ±8°, Brightness: ±0.2
Hardware (GPU)	NVIDIA RTX 3090	NVIDIA RTX 3090	NVIDIA A100 (40 GB)
Hardware (CPU)	Intel Xeon E5-2680 v4 (14 cores)	Intel Xeon E5-2680 v4 (14 cores)	Intel Xeon E5-2680 v4 (14 cores)
Training Time (hrs)	2.8	3.5	4.2
Peak Memory (GB)	11.2	14.8	28.5
Backbone Fine-tuning	Fine-tuned (ImageNet)	Fine-tuned (ImageNet)	Fine-tuned (ImageNet-21k)
View Concatenation	Channel-wise concatenation	Cross-attention fusion	Token-level fusion (BCS as distinct token)

**Table 2:** Comparative model performance.

Model	Target	MAE (kg)	RMSE (kg)	R^2^	MAPE (%)
Baseline (ResNet18 + Concat)	Live Weight	3.52	4.61	0.81	5.8
	Carcass Weight	2.15	2.84	0.85	6.2
	Fat Mass	1.42	1.88	0.76	14.5
	Lean Mass	1.95	2.45	0.79	5.1
AGFF-Net (Attention Fusion)	Live Weight	2.18	2.95	0.91	3.5
	Carcass Weight	1.45	1.92	0.93	4.1
	Fat Mass	0.94	1.25	0.88	9.6
	Lean Mass	1.32	1.76	0.89	3.4
ViT-HR (Transformers Fusion)	Live Weight	1.95	2.65	0.93	3.1
	Carcass Weight	1.28	1.70	0.95	3.6
	Fat Mass	0.85	1.12	0.91	8.7
	Lean Mass	1.15	1.55	0.92	3.0

**Table 3:** Overfitting and stability analysis (live weight MAE in kg).

Model	Training MAE (Mean ± SD)	Validation MAE (Mean ± SD)	Generalization Gap (Val—Train)	Stability Status
Baseline (ResNet18)	2.10 ± 0.15	3.52 ± 0.28	0.72 kg	Moderate Overfitting
AGFF-Net	1.65 ± 0.08	2.18 ± 0.12	0.53 kg	Stable
ViT-HR	1.52 ± 0.05	1.95 ± 0.09	0.43 kg	Highly Stable

**Table 4:** Ten-fold cross-validation results (mean ± standard deviation across 10 folds).

Model	Live Weight R^2^	Live Weight MAE (kg)	Fat Mass R^2^	Fat Mass MAE (kg)
Baseline (ResNet18)	0.78 ± 0.06	3.65 ± 0.42	0.72 ± 0.08	1.55 ± 0.22
AGFF-Net	0.89 ± 0.03	2.25 ± 0.18	0.86 ± 0.04	1.02 ± 0.11
ViT-HR	0.92 ± 0.02	1.98 ± 0.12	0.90 ± 0.03	0.88 ± 0.08

**Table 5:** Concordance Correlation Coefficient (CCC) across models and targets.

Target Variable	Baseline (ResNet18)	AGFF-Net	ViT-HR
Live Weight	0.82	0.90	0.94
Carcass Weight	0.86	0.92	0.95
Fat Mass	0.74	0.87	0.91
Lean Mass	0.78	0.88	0.92

**Table 6:** Ablation study results isolating the contribution of each component in the ViT-HR model.

Model Variant	Backbone	Multi-Task	Tabular Inputs (BCS/Size)	Fusion Strategy	Augmentation & Regularization	LW MAE ↓ (kg)	LW R^2^ ↑	FM MAE ↓ (kg)	FM R^2^ ↑
Full ViT-HR (Proposed)	ViT-Base	Yes	Yes	Token-level	Full (Aug + L2 + Dropout)	3.21	0.91	0.84	0.89
ResNet18 backbone (Baseline)	ResNet18	Yes	Yes	Token-level	Full	5.47	0.83	1.31	0.79
Single-task learning	ViT-Base	No	Yes	Token-level	Full	4.18	0.87	1.09	0.84
Image only (no tabular inputs)	ViT-Base	Yes	No	Token-level	Full	4.63	0.85	1.22	0.81
Simple concatenation fusion	ViT-Base	Yes	Yes	Feature concatenation	Full	3.89	0.88	1.01	0.86
No augmentation & regularization	ViT-Base	Yes	Yes	Token-level	None	4.52	0.84	1.18	0.80

Notes: ↓ = Lower is better; ↑ = Higher is better.

## Data Availability

Data will be available on request.
